# Supplementary material for: Peripheral bone structure, geometry, and strength and muscle density as derived from peripheral quantitative computed tomography and mortality among rural south Indian older adults
Source: PLOS Glob Public Health. 2022 Oct 4;2(10):e0000333. doi: 10.1371/journal.pgph.0000333 (PMC10022329; doi:10.1371/journal.pgph.0000333)
Supplement: S1 Data — (ZIP) [file pgph.0000333.s001.zip › Jammy_data/Dr Jammy_s Paper data_/Mediation analysis/From Bob/MediationPsychMethods/StataMacro.rtf]

The Stata macro can be accessed using:ssc paramed
