# Supplementary material for: Peripheral bone structure, geometry, and strength and muscle density as derived from peripheral quantitative computed tomography and mortality among rural south Indian older adults
Source: PLOS Glob Public Health. 2022 Oct 4;2(10):e0000333. doi: 10.1371/journal.pgph.0000333 (PMC10022329; doi:10.1371/journal.pgph.0000333)
Supplement: Attachment — Submitted filename: Response to Reviewers 6_24_22.docx.pdf [file pgph.0000333.s002.pdf]

June 24, 2022

Bert B. Little, MA, PhD, FAAAS, FRAI, FRSM, FRSPH
Academic Editor
PLOS Global Public Health

PGPH-D-22-00199R1

Peripheral bone structure, geometry, and strength and muscle density as derived from peripheral quantitative computed tomography and mortality among rural south Indian older adults.

PLOS Global Public Health

Dear Dr. Little,

Thank you for your review of our manuscript. We have addressed all the issues you noted.

Kind regards,

Jane A. Cauley

We've checked your submission and before we can proceed, we need you to address the following issues:

1. Please provide a complete Data Availability Statement in the submission form, ensuring you include all necessary access information or a reason for why you are unable to make your data freely accessible. If your research concerns only data provided within your submission, please write "All data are in the manuscript and/or supporting information files" as your Data Availability Statement. **COMPLETED**

2. Please update your online Competing Interests statement. If you have no competing interests to declare, please state: "The authors have declared that no competing interests exist." **COMPLETED**

3. Please ensure that the funders and grant numbers match between the Financial Disclosure field and the Funding Information tab in your submission form. Note that the funders must be provided in the same order in both places as well. **COMPLETED**

4. Can you please upload a copy of your revised manuscript (clean copy) that does not contain any tracked changes or highlighting. **COMPLETED**

5. We have noticed that you have uploaded Supporting Information files, but you have not included a list of legends. Please add a full list of legends for your Supporting Information files after the references list. **COMPLETED**

6. Robert M. Boudreau, Iva Miljkovic, Pawan Kumar Sharma, P S Reddy, Susan L Greenspan, and Anne B Newman are listed in the manuscript but not in your submission system. Please add the authors in Editorial Manager. **COMPLETED**
